# Limb function and quality of life after various reconstruction methods according to tumor location following resection of osteosarcoma in distal femur

**DOI:** 10.1186/1471-2474-15-453

**Published:** 2014-12-23

**Authors:** SongFeng Xu, XiuChun Yu, Ming Xu, ZhiHou Fu, Yu Chen, YuXi Sun, Qing Su

**Affiliations:** Department of Orthopaedics, General Hospital of Ji’Nan Military Region, Ji’Nan, 250031 China

**Keywords:** Osteosarcoma, Reconstruction, Limb function, Quality of life, Alcohol-inactivated autograft replantation, Articulation preservation, Prosthesis replacement

## Abstract

**Background:**

We tried to compare the functional and psychosocial outcomes after various reconstruction methods according to tumor location following resection of osteosarcoma in distal femur.

**Methods:**

We retrospectively reviewed 51 patients who underwent limb-salvage surgery of osteosarcoma in distal femur in our institution, 30 males and 21 females with an average age of 21 years (range 13–51 years). We classified osteosarcoma in distal femur into 3 types, and organized affected limb reconstruction methods after wide resection. MSTS and QOL scores were used to analyze the functional and psychological outcomes.

**Results:**

After a mean follow-up of 43 months (12–225 months), there is no difference on functional results and QOL scores among three reconstruction groups (p > 0.05) and among three types groups (p > 0.05). No difference could be noticed on tumor-free survival and total survival among three reconstruction groups (p > 0.05) and three type groups (p > 0.05). In ≤2-year, better functional scores could be found in prosthesis group, rather than the other two inactivated-bone groups (p < 0.05).

**Conclusions:**

Biological reconstruction with alcohol-inactivated autograft replantation could avoid prosthesis related complications and achieved comparable results with prosthesis following resection of osteosarcoma in distal femur. Different reconstruction options could be chosen according to tumor location, such as the distance to Insall line.

**Electronic supplementary material:**

The online version of this article (doi:10.1186/1471-2474-15-453) contains supplementary material, which is available to authorized users.

## Background

Osteosarcoma, the most common primary malignant bone tumor, usually arises in the metaphysis of long bones such as the distal femur, during the second decade of life [1]. Due to the various anatomical factors unique to juxta-articular osteosarcomas around the knee, the reconstruction in distal femur remains a challenging problem. Greater understanding of the functionality and quality of life of the osteosarcoma survivor has the potential to impact treatment decision-making and provision of follow-up services. In addition, few studies have described the application of alcohol-inactivated autograft replantation following resection of osteosarcoma in distal femur.

We classified osteosarcoma in distal femur encountered at our department into 3 types according to the location of the tumor by preoperative MRI, and organized affected limb reconstruction methods after wide resection. We performed this study to compare the functional and psychosocial outcomes of limb-salvage surgery after various reconstruction methods according to tumor location following resection of osteosarcoma in distal femur. The other purpose was to determine whether the reconstruction option of alcohol-inactivated autograft replantation could be comparable with prosthesis following resection of osteosarcoma in distal femur.

## Methods

The retrospective collection of clinical data and the publication of the data were in accordance with local guidelines for research ethics and were approved by the research ethics committee of General Hospital of Ji’Nan Military Region, Ji’Nan. All patients were treated by the same chief surgeon (XCY) and his assistants (SFX and MX) in the same institute. Written informed consent for participation in the study was obtained from all patients, including permission to access patient records and to publish individual clinical details. All procedures were in compliance with the Helsinki Declaration.

We retrospectively reviewed 51 patients who underwent limb-salvage surgery of osteosarcoma in distal femur at our institution, and were at least 12 years old at the time of operation. All patients received a diagnosis of osteosarcoma by biopsy and pathology. The surgery had been carried out between May 1995 and November 2012.

There were 30 males and 21 females with an average age of 21 years (range 13–51 years). 47 were under 30 years. One patient was with lung node and the other one with lung metastasis diagnosed by biopsy. No patients were with pathological fracture.

Preoperative chemotherapy was performed 2 times on a neoadjuvant basis. Two chemotherapy protocols were used: the DIA [2] and MMIA protocols [3]. Evidence of good chemotherapeutic response consisted of sclerotic changes or good margin of the tumor observed on plain radiographs, marked shrinkage of tumors extending into soft tissue on MR images.

### Operative treatment

Surgical technique followed standard oncologic principles of segmental resection as previously described [4]. Alcohol-inactivated autograft replantation was used in selective cases. Ligaments and tendons were reattached if possible, but this varied on a case-by-case basis. 10 patients had experienced alcohol-inactivated autograft replantation with articulation preservation, and 11 experienced alcohol-inactivated bone replantation without articulation preservation. Internal plate, screw, and intramedullary nail were used as internal fixations in inactivated autograft replantation [5]. The other 30 patients had experienced custom-made rotating hinged knee prosthesis (LiDaKang, BeiJing, China) replacement.

The conventional anterormedial incision encircling the biopsy scar for the knee was used. The surgical technique, taking the inactivated autograft replantation with articulation preservation as a sample, was described as follows [6]: (1) The lesion in distal femur was resected according to tumor-free technique rules at least 1 cm over tumor boundary, and the distal articular surface below Insall line was preserved according to preoperative MRI. (2) Then soft tissue and extraosseous tumor were cleared off. The medullar cavity was reamed and intraosseous tumor was curetted. (3) Preliminary screw fixation was prepared. The prepared autograft was then immerged into 99% alcohol for 30 minutes, retrieved and flushed with 3000 ml physiological saline. (4) The intramedullary nail was inserted into the inactivated bone off the table by carefully pressurizing cement into the inactivated bone, using the operator’s thumb to occlude the proximal medullar canal. Any excess cement was removed from the protruding stem of the femoral component and from the distal end of the inactivated bone. (5) After cylindrical reaming of the proximal femur, the intramedullary nail was inserted and cemented. (6) Before polymerization of the cement, the inactivated autograft was fixed quickly into the distal preserved articular surface with screws through previously prepared cross screw route. Care was taken so no cement was caught between the inactivated autograft and the host bone. If possible, it is recommended that autogenous iliac bone grafts were placed at the inactivated autograft-host bone junction to form extracortical grafting.

### Tumor location

**Figure 1:**
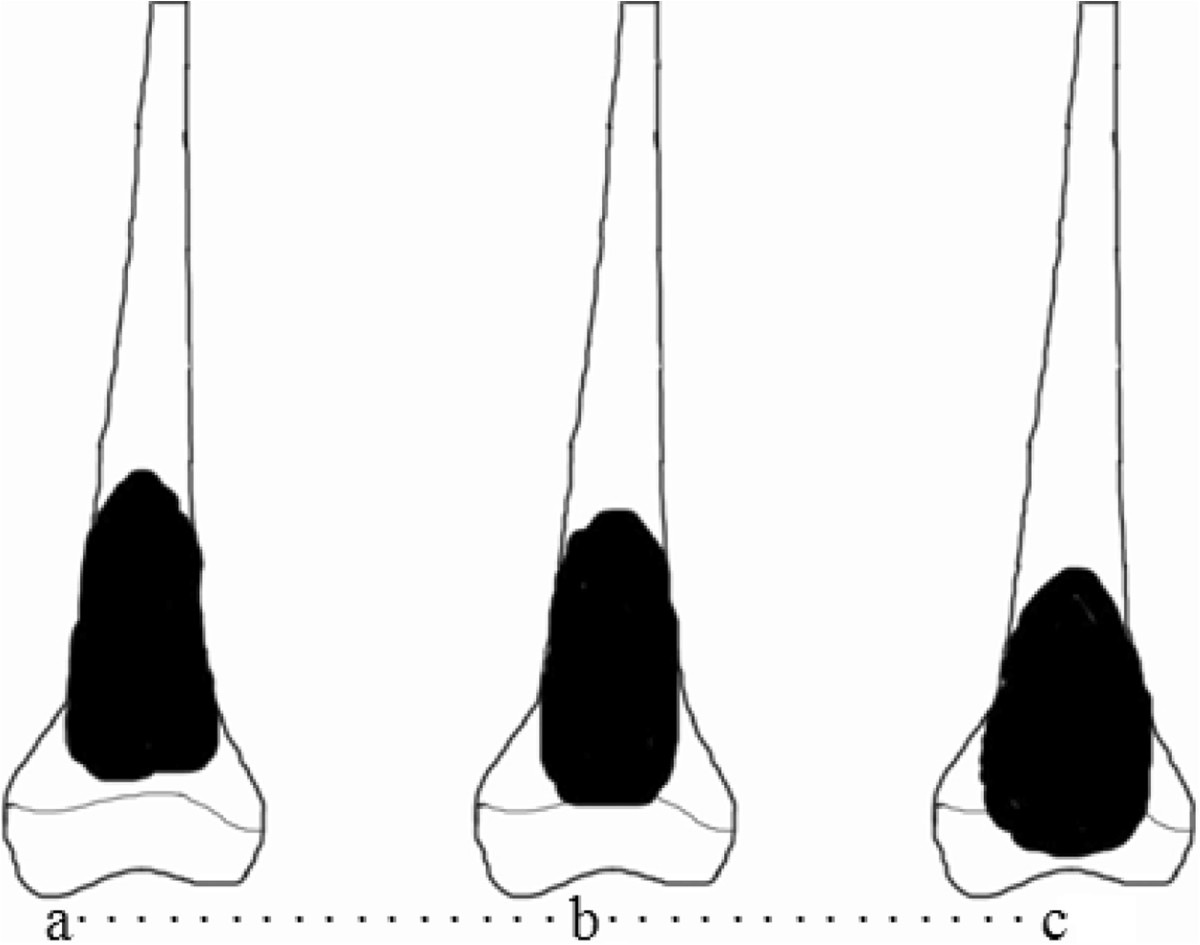
**Classification of tumor location according to preoperative MRI.** Type I **(a)**, tumors located in the diaphysis at a distance of ≥ 1 cm from the Insall line. Type II **(b)**, tumors located in contact with the Insall line or within 1 cm from this line. Type III **(c)**, tumors extended from the diaphysis to the epiphysis beyond the Insall line.

Tumor location in distal femur was classified into 3 types by the extension of osteosarcoma according to preoperative MRI (Figure 1). The extension of the tumor was evaluated on T1-weighted, T2-weighted, and Gd-enhanced T1-weighted MRI images in coronal, sagittal, and axial planes.

**Figure 2:**
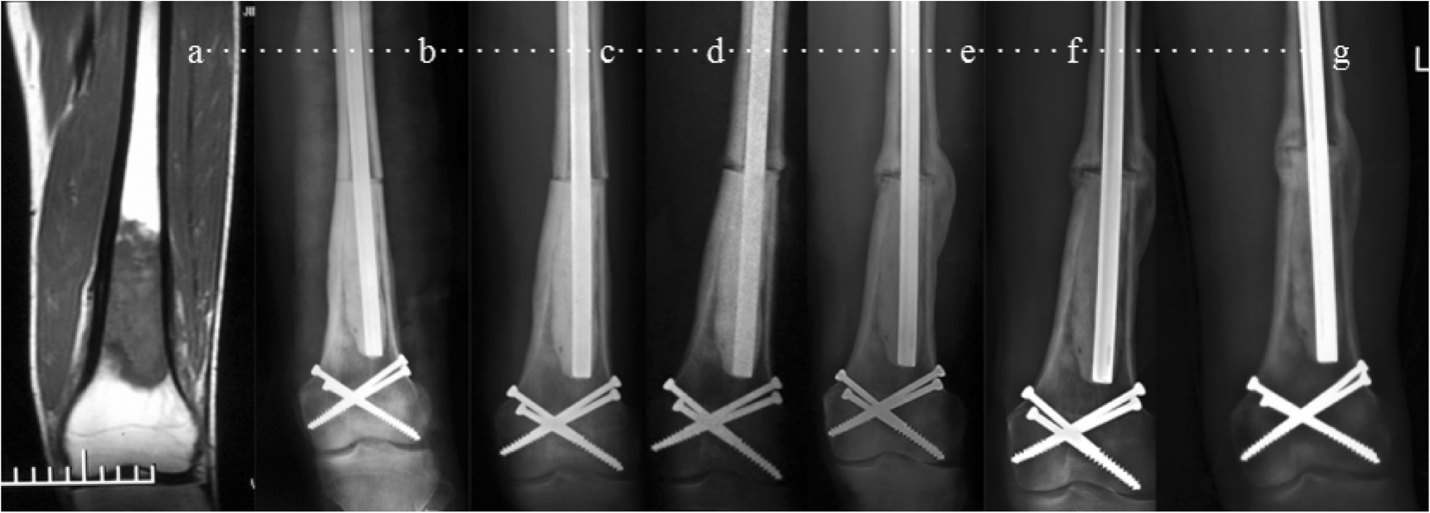
**A 20-year-old male patient with osteosarcoma in left distal femur was treated with alcohol-inactivated autograft replantation with articulation preservation. a** Preoperative MRI showed intramedullary low mixed signal in T1 in distal femur. The lowest boarding of tumor lies 2cm over Insall line which was classified as Type I. **b-d** Postoperative X-ray at 1 week, 3 months and 6 months. **e** and **f** 16 months and 20 monthes after operation, X-ray showed bone callus in diaphysis, and bony healing in conjunction between host bone and inactivated bone. **g** 35 months after operation, X-ray showed fully bony healing and good joint space. At the end of follow-up, he has returned to normal work with 33 in MSTS score and 53 in QOL.

**Figure 3:**
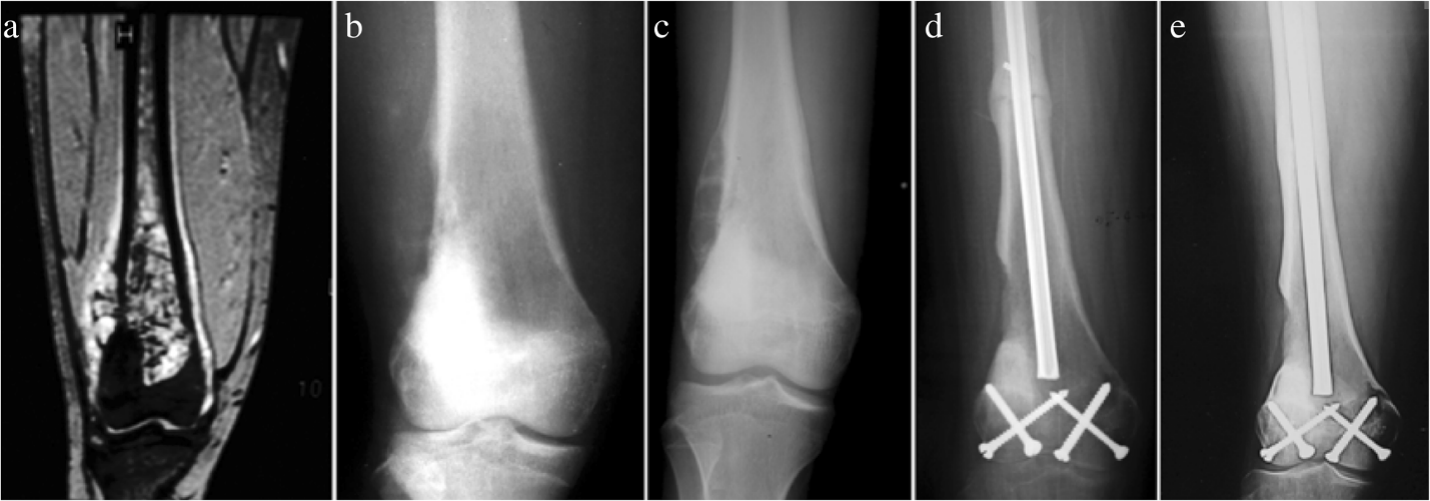
**A 15-year-old female patient with osteosarcoma in right distal femur was treated with alcohol-inactivated autograft replantation with articulation preservation. a**. Preoperative MRI showed intramedullary high and low mixed signal in distal femur. The tumor lies near Insall line which was classified as Type II. **b**. Preoperative X-ray showed osteolytic bone destruction in distal femur, in accompany with severe bone destruction in lateral cortical bone, local soft tissue mass and periosteal reaction. **c**. Postoperative X-ray showed significantly reduced osteolytic bone destruction in distal femur, in accompany with local soft tissue mass disappearance and restoration of the continuity of the lateral periosteum. **d**. Two months after operation, X-ray showed bone callus in diaphysis, and bony healing in conjunction between host bone and inactivated bone. **e**. 110 months after operation, X-ray showed fully bony healing and good joint space.

**Figure 4:**
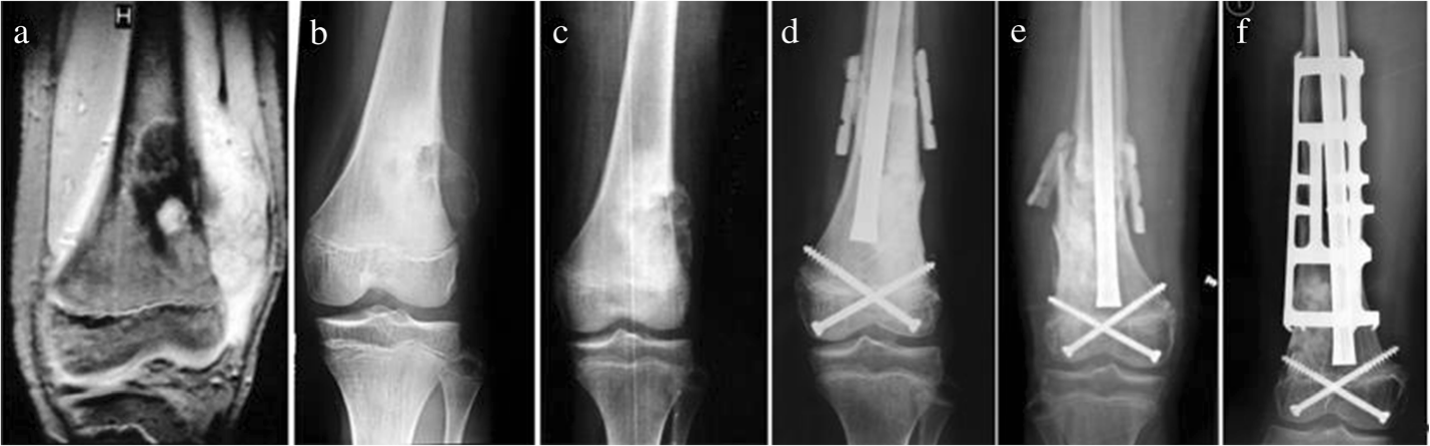
**A 15-year-old male patient with osteosarcoma in left distal femur was treated with alcohol-inactivated autograft replantation with articulation preservation. a**. Preoperative MRI showed intramedullary high and low mixed signal in distal femur. The lowest boarding of tumor lies 2cm over Insall line which was classified as Type I. **b**. Preoperative X-ray showed osteolytic bone destruction in distal femur, in accompany with severe bone destruction in lateral cortical bone, local soft tissue mass and periosteal reaction. **c**. Postoperative X-ray showed significantly reduced osteolytic bone destruction in distal femur, in accompany with local soft tissue mass disappearance and restoration of the continuity of the lateral periosteum. **d**. Two weeks after operation, X-ray showed reposition of inactivated bone with outside cortical allograft bone bridging. **e**. Nine months after operation, X-ray showed diaphysis fracture and bony healing in conjunction between host bone and inactivated bone. **f**. 30 months after reoperation, X-ray showed fully bony healing and good joint space.

**Figure 5:**
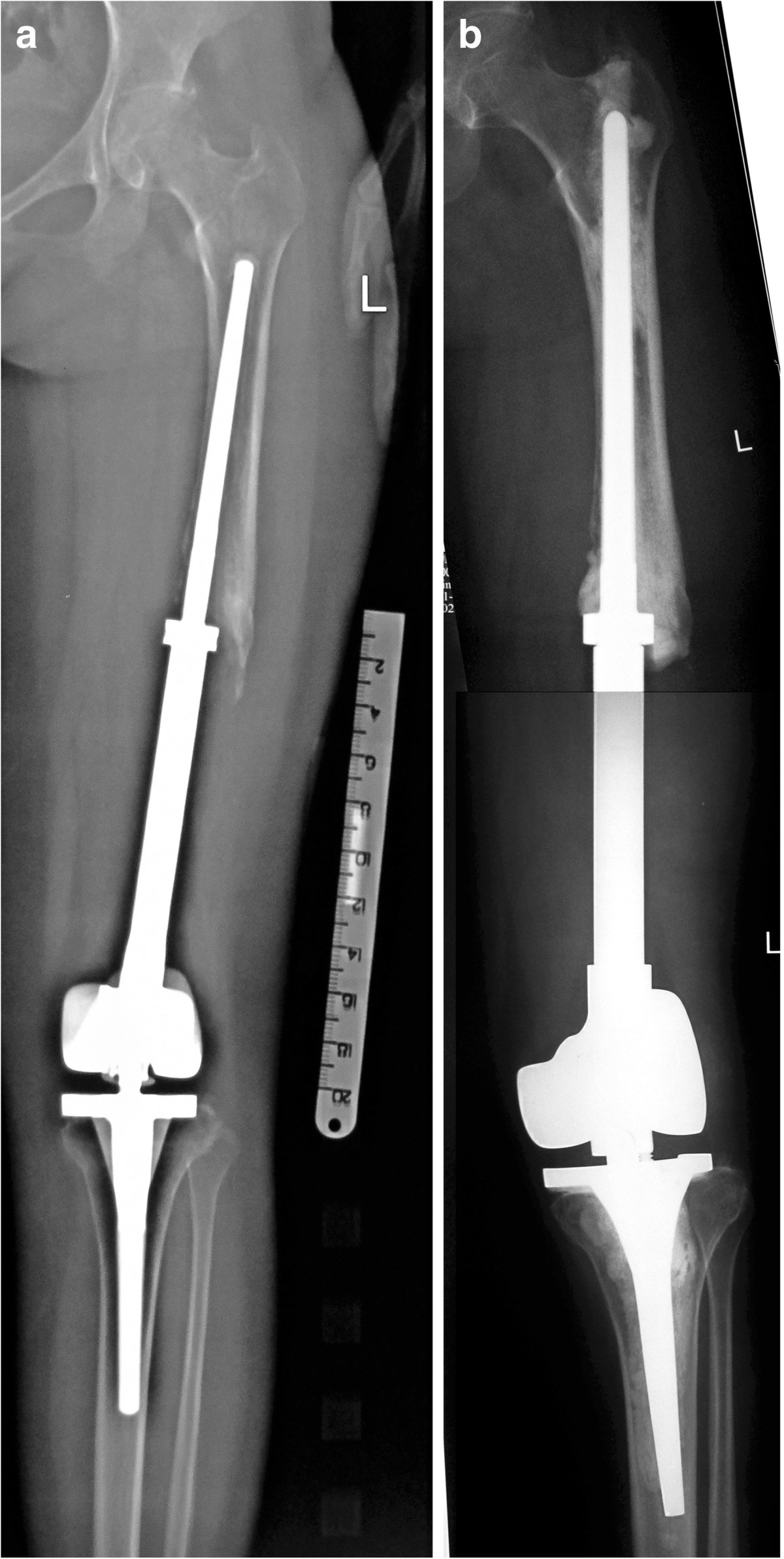
**A 17-year-old female patient with osteosarcoma in left distal femur was treated with prosthesis replacement. a** 8 years after operation, bone lose and prosthesis loosening could be found on X-ray. Physical examination demonstrated 6 cm limb length discrepancy with 26 in MSTS score and 44 in QOL. **b** After prosthesis revision, the left lower limb alignment restored and 3 cm limb length discrepancy remained.

1. Type IType I tumors were those located in the diaphysis at a distance of ≥ 1 cm from the Insall line. There were 8 patients with this type (Figure 2). Reconstruction in 7 patients was performed using inactivated autograft replantation with articulation preservation, and in 1 patient diagnosed as parosteal osteosarcoma without neoadjuvant chemotherapy using inactivated autograft replantation without articulation preservation.

2. Type IIType II tumors were those located in contact with the Insall line or within 1 cm from this line. There were 14 patients with this type (Figure 3). Reconstruction in 3 patients was performed using inactivated autograft replantation with articulation preservation (Figure 4), and in 9 patients using inactivated autograft replantation without articulation preservation. In the other 2 patients with severe bone destruction, the custom-made rotating hinged knee prosthesis was used.

3. Type IIIType III tumors were those extending from the diaphysis to the epiphysis beyond the Insall line. This type was the most frequently observed (29 patients) (Figure 5). Reconstruction was performed using the custom-made rotating hinged knee prosthesis in all patients, one of whom was with lung metastasis.

### Postoperative treatment

Prophylactic antibiotics were administered for 48 hours. Low-molecular-weight heparin was administered for 2 weeks in prosthesis group and none in inactivated-replantation group. For postoperative treatment, patients in prosthesis group were placed on bed for 1 week without external brace and in inactivated-bone group on bed for 6 weeks with external brace. After that, partial weight bearing was allowed initially, and then weight-bearing using two elbow crutches was allowed. Full weight bearing with no support was allowed at the end of 3 months in prosthesis group and 6 months in inactivated-replantation group. Plain anteroposterior and lateral radiographic examinations were done every 3 months for two years, bi-annually for a further three years and annually thereafter.

Except one who refused chemotherapy administration, all were administered chemotherapy 6 times postoperatively with two protocols: the DIA and MMIA protocols [3].

### Functional assessment

Limb function was evaluated with the Musculoskeletal Tumor Society (MSTS) rating scales, which comprise seven items, namely motion, pain, stability, deformity, strength, functional activity and emotional acceptance. The highest possible score is 35 and 5 points being allocated to each item [7]. According to the follow-up period, it was divided into ≤2-year group and >2-year group.

### Quality of life assessment

The Quality of Life (QOL)—modified specific scale for Chinese cancer patient [2] is a 12-item questionnaire that measures quality of life (appetite, sleep quality, pain, daily life condition, facial expressions, adverse reactions, fatigue, spiritual well-being, cancer knowledge, attitude to treatment, understanding and cooperation of family members and colleagues). Each item is rated on a 5-point Likert-type scale. The total score is 60. The results are ≤ 20 (very poor), 21–30 (poor), 31–40 (fair), 41–50 (good), and 51–60 (very good). High scores indicate good quality of life. According to the follow-up period, it was divided into ≤2-year group and >2-year group.

### Statistics

Summary statistics were calculated for patient characteristics by type and reconstruction groups. Chi-square tests of independence were used to assess group differences in sex and race. Median tests were used to assess group differences in age at diagnosis, duration from diagnosis to study participation, and age at the time of study participation. Means, standard deviations (SDs), and confidence intervals were calculated for each functional and psychological outcome variable by type and reconstruction groups. Multiple linear regression models were used to examine differences in functional and psychological outcomes among the three patient groups and three type groups after adjusting for duration from diagnosis to study participation.

## Results

**Figure 6:**
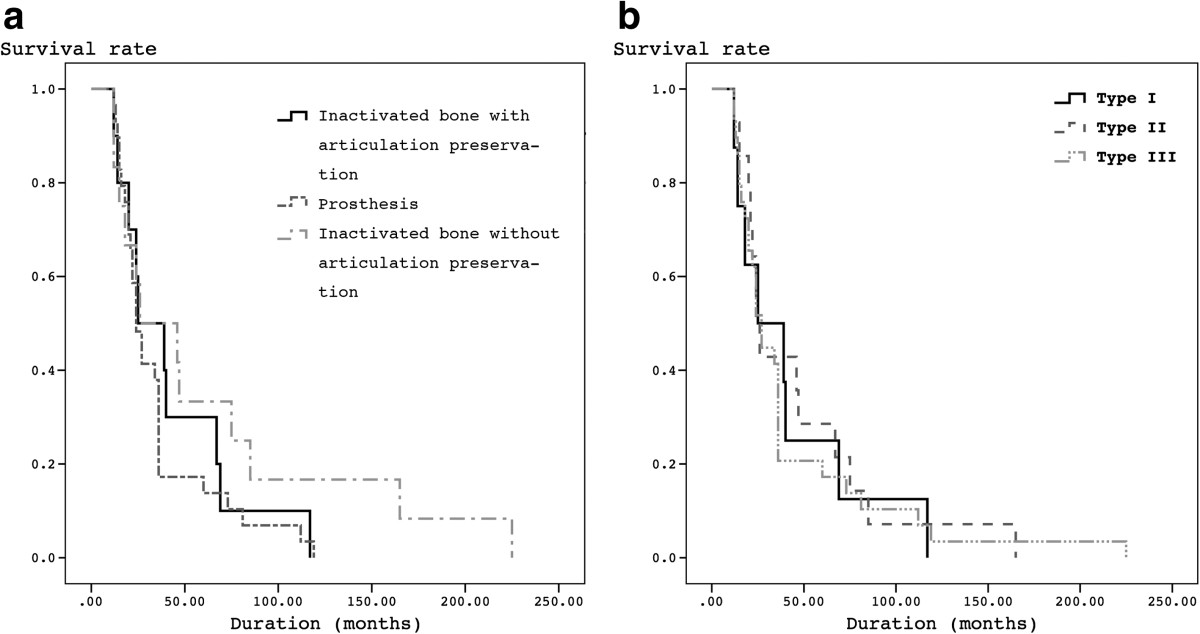
**Kaplan-Meier survival curves showing survival by 3 reconstructions (a) and by 3 types (b) in patients following resection of osteosarcoma in distal femur.** There is no difference on tumor-free survival and total survival among three reconstruction groups (p < 0.01) and three type groups (p < 0.01).

The mean follow-up was 43 months ranging from 12 to 225 months. 21 were followed for more than 2 years, and 12 for more than 5 years. 2 patients experienced inactivated-bone fracture and reoperation with internal fixation. 4 patients experienced prostheses loosening ranging from 17 to 99 months after operation in prosthesis group. 11 experienced recurrence ranging from 4 to 29 months after operation. The mean recurrence time was for 12.5 months. 23 experienced metastasis ranging from 5 to 44 months after operation. The mean metastasis time was for 19.8 months. Local infection is 5/29 (17.2%) and prosthesis loosening is 4/29(13.8%).There is no difference on functional results and QOL scores among three reconstruction groups (p > 0.05). Meanwhile, there is no difference on functional results and QOL scores among three types groups (p > 0.05). There is no difference on tumor-free survival and total survival among three reconstruction groups (p > 0.05) and three type groups (p > 0.05) (Figure 6).

In ≤2-year, better functional scores could be found in prosthesis group, rather than the other two inactivated-bone groups (p < 0.05). In >2-year, no difference in functional and QOL scores could be found among three groups (p > 0.05). However, lower incidence of prosthesis related complications could be found in inactivated-replantation group, whatever articulation preservation. No relationship could be found between functional results and QOL (p > 0.05) whenever in ≤2-year group or in >2-year group.

Reconstruction after tumor resection was performed by inactivated-bone replantation with articulation preservation in 10 patients. There were 7 males and 3 females, ranging in age from 15 to 34 years (mean, 21 years). Tumors were Type I in 7 patients, Type II in 3 patients. Two experienced inactivated bone fracture and reoperation with internal fixation 7 and 8 months after operation respectively. Recurrence occurred in 1 patient. One experienced arthroscopic release for knee stiffness. At the end of follow-up, 6 patients had died due to multiple metastases after a mean of 14 months, ranging from 5 to 29 months. The MSTS score in ≤2-year is 22.6 (63%), and in >2-year is 26.3 (73%). QOL in ≤2-year is 28.8, and in >2-year is 40.8.

Reconstruction was performed by alcohol-inactivated autograft replantation without articulation preservation in 12 patients. There were 6 males and 6 females, ranging in age from 14 to 34 years (mean, 19 years). Tumors were Type I in 1 patient, Type II in 9 patients and Type III in 2 patients. No inactivated bone fracture occurred. Recurrence occurred in 5 patients who experienced metastases lately, ranging from 7 to 16 months (mean, 12.2 months). 2 were treated with custom-made rotating hinged knee prosthesis replacement and hip disarticulation respectively. The MSTS score in ≤2-year is 23.3 (64%), and in >2-year is 28.2 (78%). QOL in ≤2-year is 30.8, and in >2-year is 42.3.

Reconstruction was performed by custom-made rotating hinged knee prosthesis reconstruction in 29 patients. There were 18 males and 11 females, ranging in age from 13 to 51 years (mean, 21 years). Tumors were Type II in 2 patients with severe bone destruction and Type III in other 27 patients. Prosthesis loosening occurred in 4 patients. Infection occurred in 5 patients, 2 of who experienced skin ulcer and reoperation of muscle flap transposition. Recurrence occurred in 6 patients. At the end of follow-up, 12 patients had died due to multiple metastases after a mean of 26.5 months, ranging from 6 to 44 months. The MSTS score in ≤2-year is 27.6 (76%), and in >2-year is 26.7 (74%). QOL in ≤2-year is 46.8, and in >2-year is 45.4.

## Discussion

When the affected limb is reconstructed after the resection of a malignant tumor in adolescence, such reconstruction is associated with a variety of problems, including an expectation of joint function due to postoperative articulation preservation, measures to be taken to cope with high levels of physical activity, and problems related to social adaptation. In other side, there are various limb reconstruction methods for malignant bone tumors in distal femur, and each method has advantages and disadvantages. There is no optimal good reconstruction method for each patient.To solve these problems, we attempted to classify reconstruction of the lower limbs into 3 types based on the sites of tumor location in distal femur on MRI (Figure 1). This classification served as a guide for the resection margins and the available choice of different reconstruction methods of in distal femur. Type I tumors were those located in the diaphysis. Type II tumors were those in contact with the Insall line, and Type III tumors were those infiltrating the epiphysis through the Insall line. In our opinion, the first type involves reconstruction of the long bone shaft, alcohol-inactivated autograft replantation with articulation preservation is recommended to be the most useful technique. This approach is apparently applicable to reconstruction of the diaphysis, and has less influence on limb function associated with malignant bone tumor resection in distal femur. For Type II tumors, which are located in the diaphysis in contact with Insall line, when adjunctive therapies such as chemotherapy are effective, there is a chance of preserving the articulation. If impossible, alcohol-inactivated autograft replantation without articulation preservation is recommended, which may results in joint surface degeneration and joint instability. The third type includes tumors invading the epiphyses that require an adequately wide resection with margins of at least 3 cm in the surrounding tissue. This type is an indication for wide resection and prosthesis replacement. The results showed that this classification can be successfully used as a guide for appropriate decision-making in all cases following resection of osteosarcoma in distal femur.

According to Manfrini’s [8] report, if preoperative MRI demonstrated no invasion of malignant tumor into epiphysis, the limb-salvage surgery with epiphysis preservation should be considered for better limb function. Similarly, Yoshida et al. [9] classified reconstruction of the lower limbs in pediatric malignant bone tumors into 3 types according to preoperative MRI, and emphysized that a limb reconstruction method allowing the maximal preservation of joint function should be selected after careful evaluation of the effects of chemotherapy and the location of the tumor. The reason why we chose Insall line were that : first, Insall line lies near epiphyseal line; second, it is easy for intraoperative localization; third, it would be convenient for preservation of lateral and medial collateral ligament which do good for knee joint instability. In our previous management of 7 malignant bone tumor around knee, it was showed that alcohol-inactivated autograft replantation with articulation preservation could improve the short-term limb function, avoiding complications with prosthesis [10].

Neoadjuvant chemotherapy is the breakthrough for treatment of osteosarcoma, which could destroy the primary tumor and achieve safe surgical resections. Disease free survival was escalated from <20% prior to the introduction of effective chemotherapy to 55–75% and overall survival to 85% [11]. Further, the opportunity to perform limb salvage was expanded to 80% of patients [11]. The introduction of chemotherapy to the treatment of osteosarcoma caused a paradigm shift in surgical procedure [12]. Efficient chemotherapy has made long survival possible after excision of osteosarcoma and has helped to minimize the surgical margins. After effective preoperative chemotherapy, osteosarcoma can be excised with the preservation of a maximum of healthy tissue, such as ligaments and tendons. In our previous practice, with careful preoperative evaluation and effective preoperative chemotherapy marginal resection of osteosarcoma can produce good results [3]. On this consideration, 3 patients of Type II experienced marginal resection and inactivated autograft replantation with articulation preservation, after careful evaluation of good chemotherapeutic response.

Achieving complete ablation of the tumor and preserving a functional extremity at the same time proves to be a difficult task due to the various anatomical factors unique to distal femur. The most popular reconstructive methods are prosthesis and biological reconstruction. Prosthesis has been recommended as an optimal reconstruction choice after resection of juxta-articular osteosarcoma around the Knee [13]. Prosthesis failures are classified as soft-tissue failures, aseptic loosening, structural failures, infection, and tumor progression [14]. Deep infection is the most serious of these complications and the infection rate for patients undergoing endoprosthetic implantation is between 3.6% and 44.6% [14, 15]. In this study, local infection is 5/29 (17.2%) and prosthesis loosening is 4/29 (13.8%). There is no difference among three type groups and three reconstruction groups in over-2-year on functional results and QOL. While in inactivated-bone group whenever articulation preservation, no infection occurred. However, none of prosthesis related complications could be found in inactivated autograft group, whatever articulation preservation, demonstrated that biological reconstruction with inactivated-bone could avoid prosthesis related complications and achieved comparable results with prosthesis. This reconstruction method is a feasible option following resection of osteosarcoma in distal femur.

At present, the most popular biological reconstruction method following skeletal tumor resection is allografting [16–19]. However, there are problems relating to infectious transmission, immunological reaction and refusal based on social or religious beliefs especially in Asian countries. Allograft reconstruction for extremity sarcomas had a high rate of adverse events (70%) and allograft removal (60%) in patients followed for at least 10 years [20]. Under these circumstances, recycled tumor bone autografts are widely used as an alternative to bone allografts. Devitalized bone autograft is particularly well suited in the region where allografts are not readily available [21]. Techniques that are capable of destroying tumor cells in resected bone include (1) irradiation [17, 19], (2) autoclaving [22], (3) pasteurization [2, 23], (4) freezing-thawing with liquid nitrogen [24] and alcohol inactivation [25, 26].

Compared to other methods, alcohol inactivation method is considered on the same level of safety in oncological control, which superiorities are economic-applicable to patients and the well fitness of bone graft with the defect. The disadvantage of alcohol-inactivated autograft is that it needs a long time to accomplish revascularization and to integrate with surrounding bone. The rationality of alcohol inactivation is that alcohol could devitalize the tumor bone shell. The tumor cells had been devitalized when the ingrowth of surrounding vessels occurred [5]. Our previous studies showed that continuous bone callus presented after 8 weeks and complete bony healing showed after 12 weeks in rabbit femur [27]. The irradiated allograft presented the likely bony healing process of creeping substitution with the bone formation rate of 1 cm per 10 months [21]. In our previous study on alcohol- inactivated autograft, according to the dynamic imaging observation and ISOLS composite scoring, the new bone originated from host bone and the bone healing time in femur is about 4 to 6 months and that in tibia about 6–8 months [6]. We hold the viewpoint that creeping substitution is possibly the main way in bony junction and the healing time in femur is faster than that in tibia.

As we known, there is no reports concerned the joint function and QOL between alcohol-inactivated replantation alone and mega-prosthesis. Biological reconstruction, using distraction osteogenesis or frozen autografts, could yield good functional results and QOL without leading to an increase in the incidence of local recurrence [28]. After retrospective review of 20 patients who underwent primary osteoarticular allograft reconstruction after extremity sarcoma resection, it was concluded that functional outcomes of patients with intact osteoarticular allografts were comparable to outcomes with prostheses replacement [20]. In this study, the results showed that prosthesis could achieve better functional results than alcohol-inactivated autograft in less than 2 years. In over 2 years, there is no difference on joint function and QOL. Alcohol-inactivated replantation could result in comparable clinical outcomes with mega-prostheses.

The emotional well-being in bone cancers patient populations has received minimal attention [29]. Robert et al. [29] reported that better leg function was significantly related to better emotional functioning in long-term osteosarcoma survivors. O’Malley et al. [30] examined 115 pediatric cancer survivors and did not find a relationship between physical function and psychological adjustment following cancer. In this study, no relationship could be found between functional results and QOL. Other limitations in this study are, small amount of patients, the less than 10 years’ follow-up, retrospective study design, variety among tumor size and use of chemotherapy protocols, which might result in biased result. The multi-centric randomized controlled study with large samples should be considered.

## Conclusion

Biological reconstruction with alcohol-inactivated autograft could avoid prosthesis related complications and achieved comparable results with prosthesis. This reconstruction method is a feasible option following resection of osteosarcoma in distal femur. Different reconstruction options could be chosen according to tumor location, such as the distance to Insall line. If possible, the reconstruction allowing the maximal articulation preservation should be selected after careful evaluation of the effects of chemotherapy and the location of the tumor.

## Authors’ original submitted files for images

Below are the links to the authors’ original submitted files for images. Authors’ original file for figure 1 Authors’ original file for figure 2 Authors’ original file for figure 3 Authors’ original file for figure 4 Authors’ original file for figure 5 Authors’ original file for figure 6

## References

[1] Ando K, Heymann MF, Stresing V, Mori K, Redini F, Heymann D (2013). Current therapeutic strategies and novel approaches in osteosarcoma. Cancers.

[2] Xu M, Xu SF, Yu XC (2014). Clinical analysis of osteosarcoma patients treated with high-dose methotrexate-free neoadjuvant chemotherapy. Curr Oncol.

[3] Yu X, Xu M, Song R, Xu S (2009). Marginal resection for osteosarcoma with effective preoperative chemotherapy. Orthop Surg.

[4] Enneking WF, Spanier SS, Goodman MA (1980). A system for the surgical staging of musculoskeletal sarcoma. Clin Orthop Relat Res.

[5] Yu XC, Liu XP, Zhou Y, Fu ZH, Song RX, Sun HN, Xu M (2007). Inactivated bone replantation with preservation of the epiphysis for osteosarcoma in children. Orthop J China.

[6] Xu S, Yu X, Xu M, Fu Z (2013). Inactivated autograft–prosthesis composite have a role for grade III giant cell tumor of bone around the knee. BMC Musculoskelet Disord.

[7] Enneking WF, Dunham W, Gebhardt MC, Malawar M, Pritchard DJ (1993). A system for the functional evaluation of reconstructive procedures after surgical treatment of tumors of the musculoskeletal system. Clin Orthop Relat Res.

[8] Manfrini M, Gasbarrini A, Malaguti C, Ceruso M, Innocenti M, Bini S, Capanna R, Campanacci M (1999). Intraepiphyseal resection of the proximal tibia and its impact on lower limb growth. Clin Orthop Relat Res.

[9] Yoshida Y, Osaka S, Tokuhashi Y (2010). Analysis of limb function after various reconstruction methods according to tumor location following resection of pediatric malignant bone tumors. World J Surg Oncol.

[10] Xu SF, Yu XC, Xu M, Liu XP, Song RX, Fu Z (2012). Surgical technique of alcohol-inactivated autograft replantation with articulation preservation in management of malignant bone tumor around knee (In Chinese). Chin J Joint Surg (Electronic Edition).

[11] Jaffe N (2009). Osteosarcoma: review of the past, impact on the future. The American experience. Cancer Treat Res.

[12] Yamamoto N, Tsuchiya H (2013). Chemotherapy for osteosarcoma - where does it come from? What is it? Where is it going?. Expert Opin Pharmacother.

[13] DiCaprio MR, Friedlaender GE (2003). Malignant bone tumors: limb sparing versus amputation. J Am Acad Orthop Surg.

[14] Henderson ER, Groundland JS, Pala E, Dennis JA, Wooten R, Cheong D, Windhager R, Kotz RI, Mercuri M, Funovics PT, Hornicek FJ, Temple HT, Ruggieri P, Letson GD (2011). Failure mode classification for tumor endoprostheses: retrospective review of five institutions and a literature review. J Bone Joint Surg Am Vol.

[15] Morii T, Morioka H, Ueda T, Araki N, Hashimoto N, Kawai A, Mochizuki K, Ichimura S (2013). Deep infection in tumor endoprosthesis around the knee: a multi-institutional study by the Japanese musculoskeletal oncology group. BMC Musculoskelet Disord.

[16] Gilbert NF, Yasko AW, Oates SD, Lewis VO, Cannon CP, Lin PP (2009). Allograft-prosthetic composite reconstruction of the proximal part of the tibia. An analysis of the early results. J Bone Joint Surg.

[17] Biau DJ, Dumaine V, Babinet A, Tomeno B, Anract P (2007). Allograft-prosthesis composites after bone tumor resection at the proximal tibia. Clin Orthop Relat Res.

[18] Donati D, Colangeli M, Colangeli S, Di Bella C, Mercuri M (2008). Allograft-prosthetic composite in the proximal tibia after bone tumor resection. Clin Orthop Relat Res.

[19] Wunder JS, Leitch K, Griffin AM, Davis AM, Bell RS (2001). Comparison of two methods of reconstruction for primary malignant tumors at the knee: a sequential cohort study. J Surg Oncol.

[20] Ogilvie CM, Crawford EA, Hosalkar HS, King JJ, Lackman RD (2009). Long-term results for limb salvage with osteoarticular allograft reconstruction. Clin Orthop Relat Res.

[21] Muramatsu K, Ihara K, Miyoshi T, Yoshida K, Iwanaga R, Hashimoto T, Taguchi T (2012). Stimulation of neo-angiogenesis by combined use of irradiated and vascularized living bone graft for oncological reconstruction. Surg Oncol.

[22] Harrington KD, Johnston JO, Kaufer HN, Luck JV, Moore TM (1986). Limb salvage and prosthetic joint reconstruction for low-grade and selected high-grade sarcomas of bone after wide resection and replacement by autoclaved [corrected] autogeneic grafts. Clin Orthop Relat Res.

[23] Jeon DG, Kim MS, Cho WH, Song WS, Lee SY (2007). Pasteurized autograft-prosthesis composite for distal femoral osteosarcoma. J Orthop Sci.

[24] Tsuchiya H, Wan SL, Sakayama K, Yamamoto N, Nishida H, Tomita K (2005). Reconstruction using an autograft containing tumour treated by liquid nitrogen. J Bone Joint Surg (Br).

[25] Yu X, Liu X, Zhou Y, Li K, Qu Z (2005). Inactivated bone replantation with preservation of the epiphysis in children with osteosarcoma: clinical report of two cases. Chin-Ger J Clin Oncol.

[26] Sung HW, Wang HM, Kuo DP, Hsu WP, Tsai YB (1986). EAR method: an alternative method of bone grafting following bone tumor resection (a preliminary report). Semin Surg Oncol.

[27] Yu XC, Liu XP, Zhou Y, Li KH, Song RX, You MR (2004). Influence of alcoholic devitalization and replantation with inactivated bone on knee function: clinical and experimental study. Chin J Bone Tumor Bone Dis.

[28] Tsuchiya H, Abdel-Wanis ME, Tomita K (2006). Biological reconstruction after excision of juxta-articular osteosarcoma around the knee: a new classification system. Anticancer Res.

[29] Robert RS, Ottaviani G, Huh WW, Palla S, Jaffe N (2010). Psychosocial and functional outcomes in long-term survivors of osteosarcoma: a comparison of limb-salvage surgery and amputation. Pediatr Blood Cancer.

[30] O’Malley JE, Foster D, Koocher G, Slavin L (1980). Visible physical impairment and psychological adjustment among pediatric cancer survivors. Am J Psychiatry.

[CR31] The pre-publication history for this paper can be accessed here:http://www.biomedcentral.com/1471-2474/15/453/prepub

