# Supplementary figures and images for: Limb function and quality of life after various reconstruction methods according to tumor location following resection of osteosarcoma in distal femur
Source: BMC Musculoskelet Disord. 2014 Dec 23;15:453. doi: 10.1186/1471-2474-15-453 (PMC4364625; doi:10.1186/1471-2474-15-453)

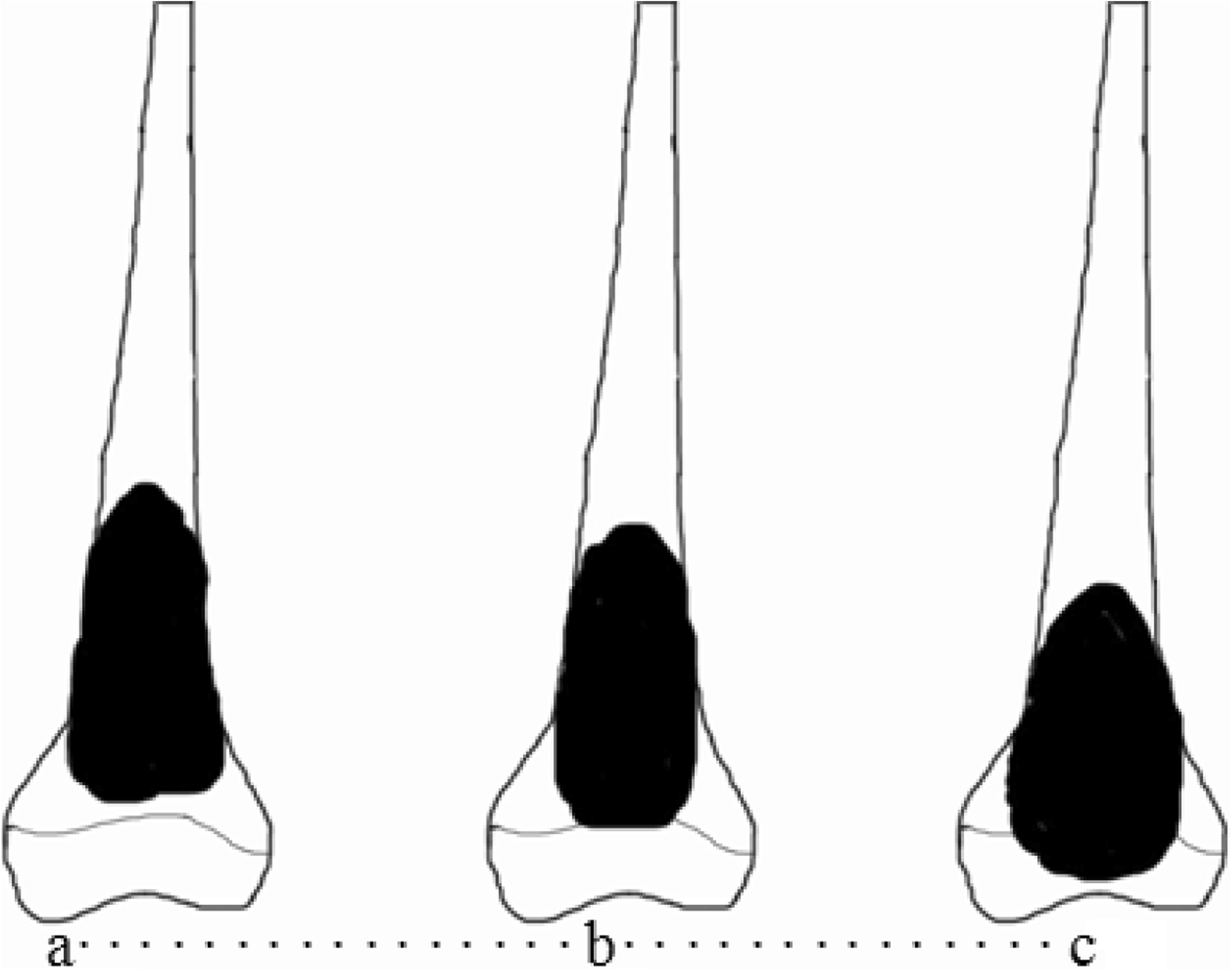

Supplement: Supplementary file 1 — Authors’ original file for figure 1 [file 12891_2014_2390_MOESM1_ESM.tif]

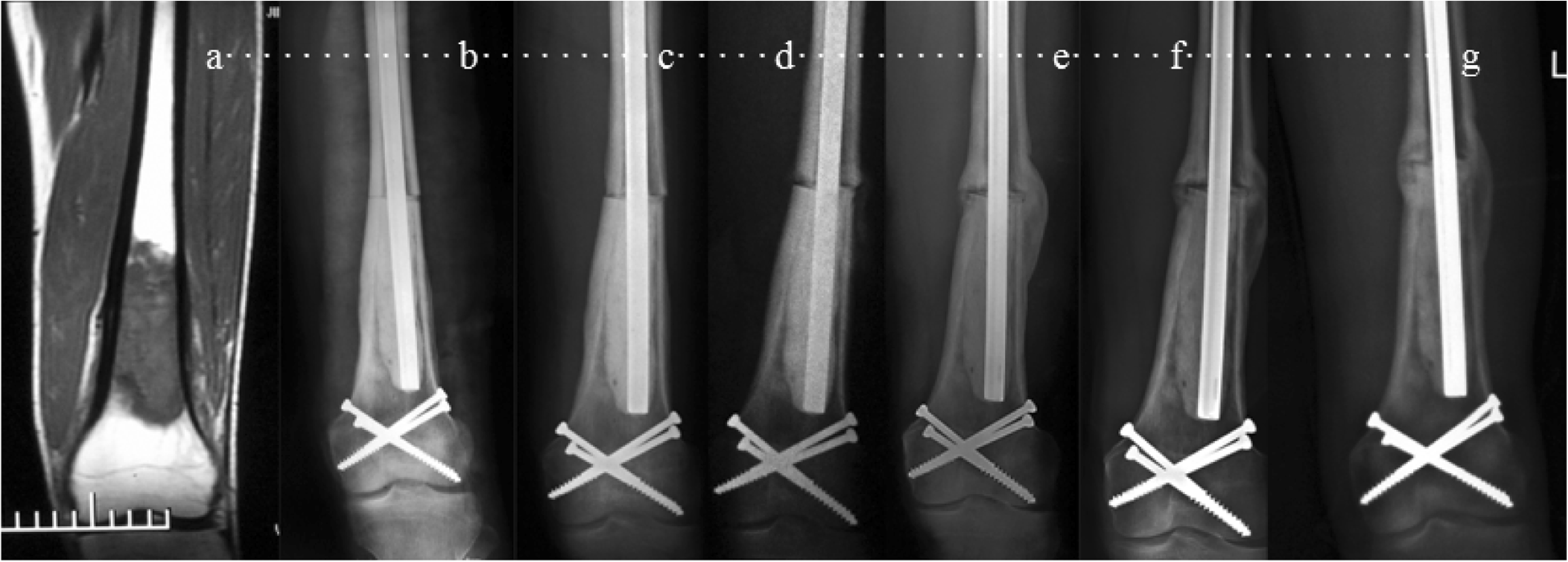

Supplement: Supplementary file 2 — Authors’ original file for figure 2 [file 12891_2014_2390_MOESM2_ESM.tif]

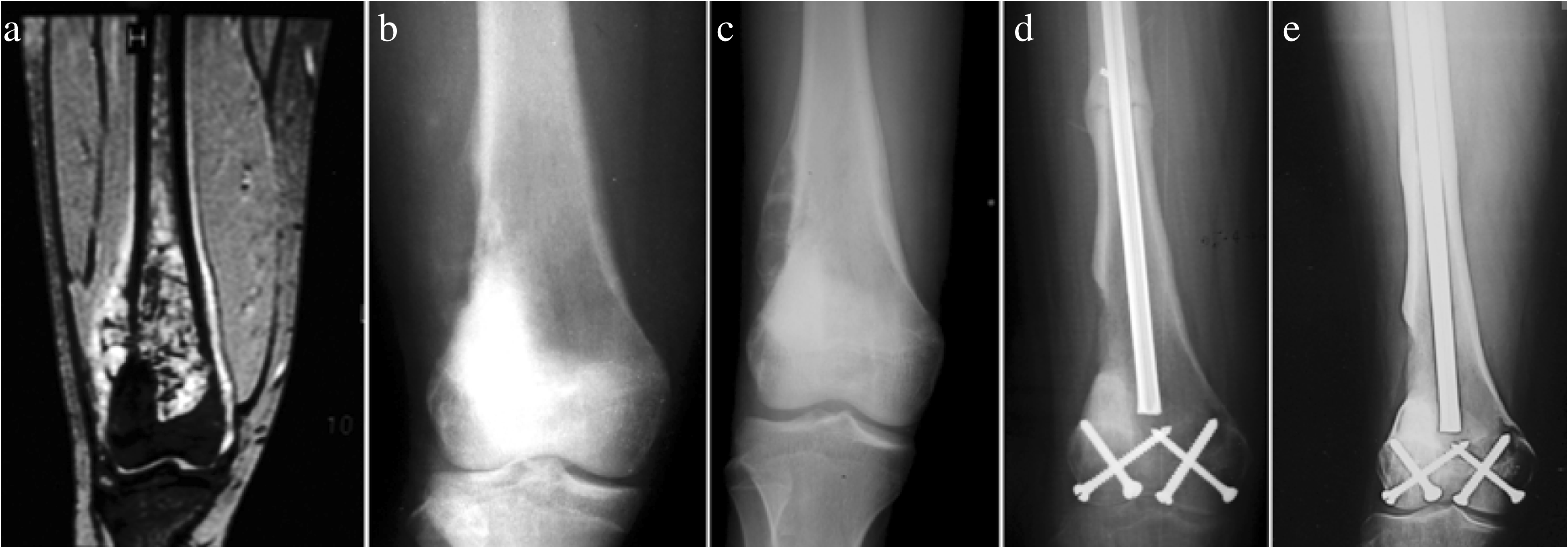

Supplement: Supplementary file 3 — Authors’ original file for figure 3 [file 12891_2014_2390_MOESM3_ESM.tif]

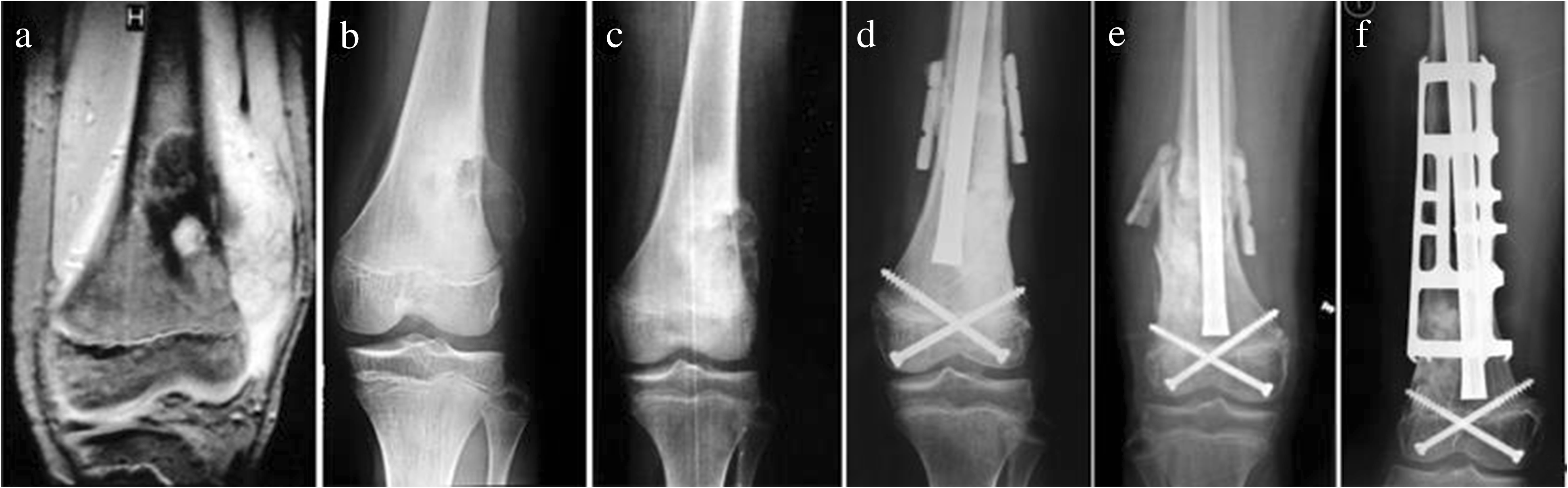

Supplement: Supplementary file 4 — Authors’ original file for figure 4 [file 12891_2014_2390_MOESM4_ESM.tiff]

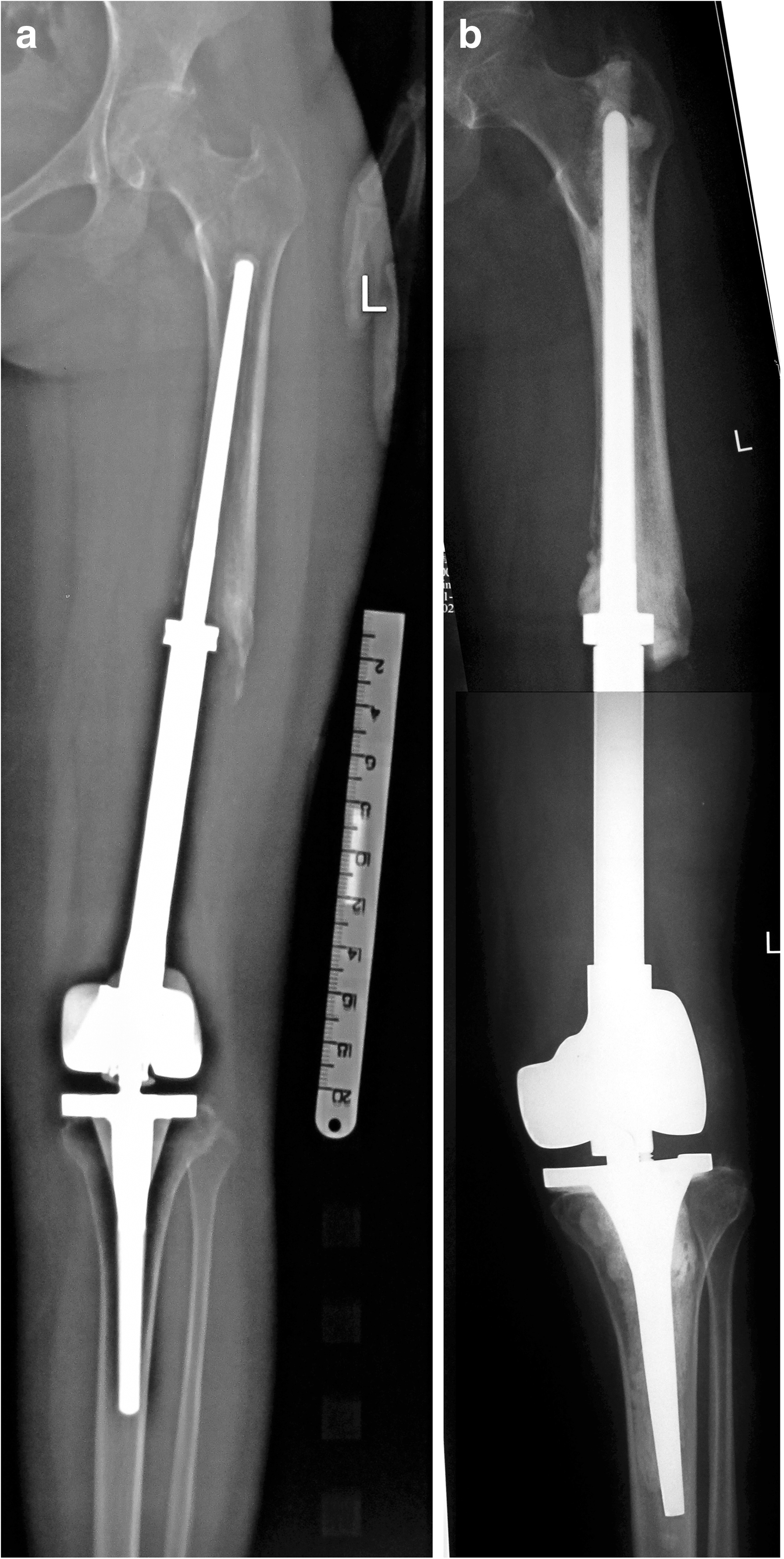

Supplement: Supplementary file 5 — Authors’ original file for figure 5 [file 12891_2014_2390_MOESM5_ESM.tiff]

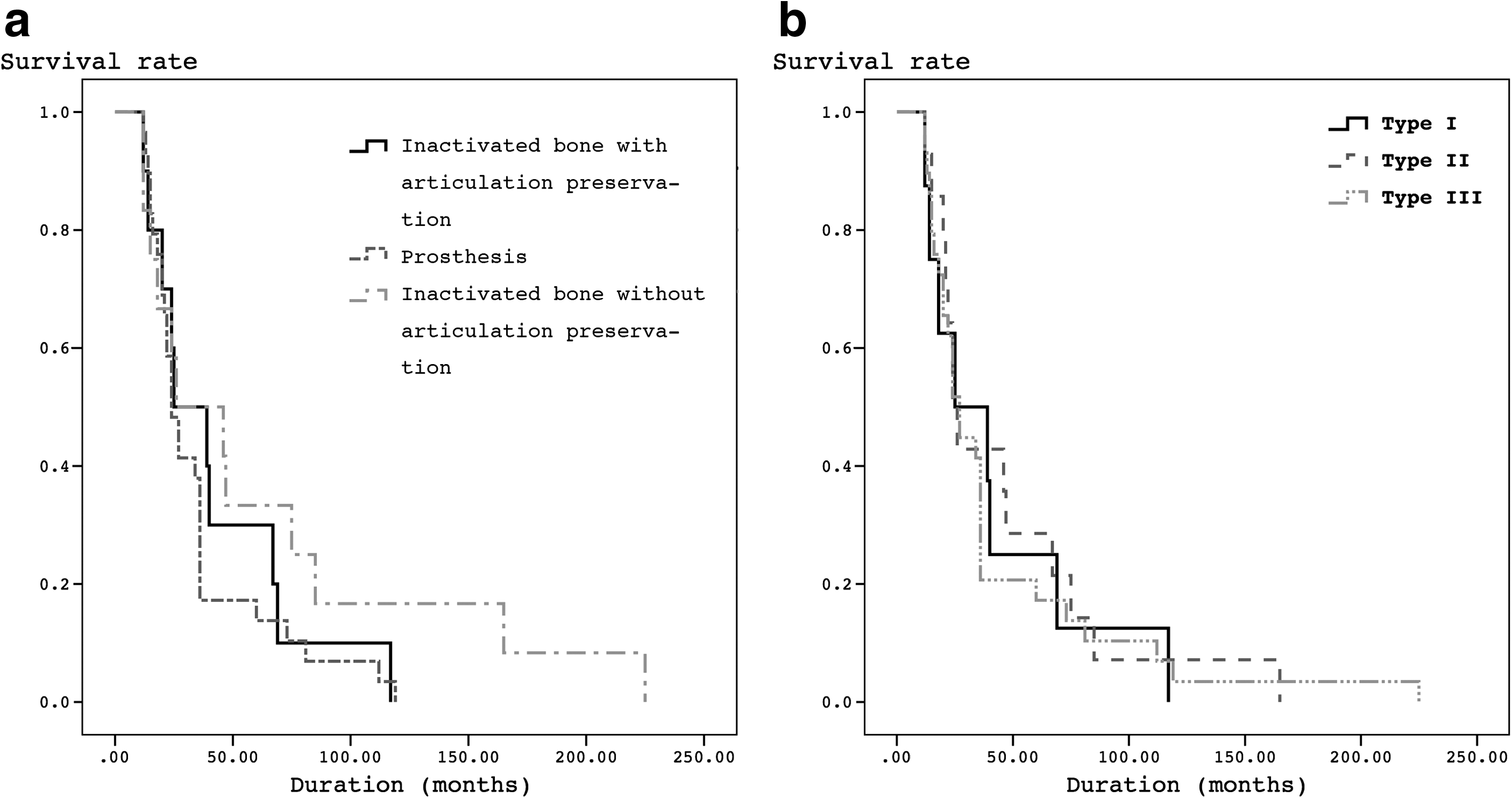

Supplement: Supplementary file 6 — Authors’ original file for figure 6 [file 12891_2014_2390_MOESM6_ESM.tiff]
